# Burning and graphitization of optically levitated nanodiamonds in vacuum

**DOI:** 10.1038/srep21633

**Published:** 2016-02-22

**Authors:** A. T. M. A. Rahman, A. C. Frangeskou, M. S. Kim, S. Bose, G. W. Morley, P. F. Barker

**Affiliations:** 1 Department of Physics and Astronomy, University College London, Gower Street, WC1E 6BT, UK; 2 Department of Physics, University of Warwick, Gibbet Hill Road, CV4 7AL, UK; 3 QOLS, Blackett Laboratory, Imperial College London, SW7 2BW, UK

## Abstract

A nitrogen-vacancy (NV^−^) centre in a nanodiamond, levitated in high vacuum, has recently been proposed as a probe for demonstrating mesoscopic centre-of-mass superpositions and for testing quantum gravity. Here, we study the behaviour of optically levitated nanodiamonds containing NV^−^ centres at sub-atmospheric pressures and show that while they burn in air, this can be prevented by replacing the air with nitrogen. However, in nitrogen the nanodiamonds graphitize below ≈10 mB. Exploiting the Brownian motion of a levitated nanodiamond, we extract its internal temperature (*T*_*i*_) and find that it would be detrimental to the NV^−^ centre’s spin coherence time. These values of *T*_*i*_ make it clear that the diamond is not melting, contradicting a recent suggestion. Additionally, using the measured damping rate of a levitated nanoparticle at a given pressure, we propose a new way of determining its size.

Even though diamond is thermodynamically metastable in ambient conditions, it has extremely high thermal conductivity, Young’s modulus, electrical resistivity, chemical stability, and optical transparency^1^,^2^,^3^,^4^. Nanodiamonds inherit most of these spectacular properties from their bulk counterparts and the inclusion of color centres such the NV^−^ centre has increased their realm of applications^1^,^5^. Proposed and demonstrated applications of diamond, nanodiamonds and nanodiamonds with NV^−^ centres include tribology^1^,^6^, nanocomposites^7^, UV detection in space applications^8^, magnetometry^9^, biological imaging^10^, quantum information processing^11^,^12^ and thermometry^13^. More recently nanodiamonds with NV^−^ centres have been suggested for testing quantum gravity^14^ and for demonstrating centre of mass (CM) superpositions of mesoscopic objects^15^,^16^. These superpositions and interferometry also point towards a broader future application of levitated diamonds in sensing and gravitometry. In the scheme for testing quantum gravity, an NV^−^ centre in a nanodiamond is exploited in a Ramsey-Borde interferometer^14^ and, in the non-relativistic limit, the first order correction to the energy dispersion scales with the size of a nanodiamond. In the case of creating CM superpositions, the NV^−^ centre’s spin is utilized and the spatial separation of the superposed CM states depends on the size of a nanodiamond^15^,^16^. To prevent the adverse effects of motional decoherence, these proposals^14^,^15^,^16^ have been conceptualized in high vacuum (10^−6^ mB). It is, however, well known that at atmospheric temperature and pressure graphite is the most stable form of carbon both in the bulk as well as at the nanoscale (>5.2 nm)^3^,^4^,^17^,^18^,^19^ while diamond is stable between ≈1.9 nm and ≈5.2 nm^17^. Since the utility of diamond and diamond with various color centres depends on its crystalline existence, it is imperative to study the behaviour of diamond in vacuum for scientific as well as for practical purposes. Furthermore, while the determination of the size of nanoparticles using electron microscopy and dynamic light scattering are well established, their utility in levitated experiments is limited if not completely excluded. As a result it seems reasonable to devise a way by which one can determine the size of an individual levitated object while performing the experiment. This is particularly useful in experiments in which the size of a nanoparticle plays important roles. The significance of *in situ* size determination is further emphasized by the polydisperse nature of nanoparticles.

In this article, we levitate high pressure high temperature (HPHT) synthesized nanodiamonds containing ≈500 NV^−^ centres (ND-NV-100 nm, Adamas Nanotechnology, USA) using an optical tweezer and study their behaviour under different levels of vacuum. We show that as the pressure of the trapping chamber is reduced, the internal temperature (*T*_*i*_) of a trapped nanodiamond can reach ≈800 K. Due to this elevated temperature levitated nanodiamonds burn in air. We also demonstrate that the burning of nanodiamond is preventable under a nitrogen environment down to 10 mB, but beyond that, it graphitizes. The source of heating is believed to be the absorption of 1064 nm trapping laser light by the impurities in diamond and the amorphous carbon on the surface. Lastly, exploiting the measured damping rate of a levitated object, we present a new way of determining its size *in situ*.

## Experimental Setup

**Figure 1:**
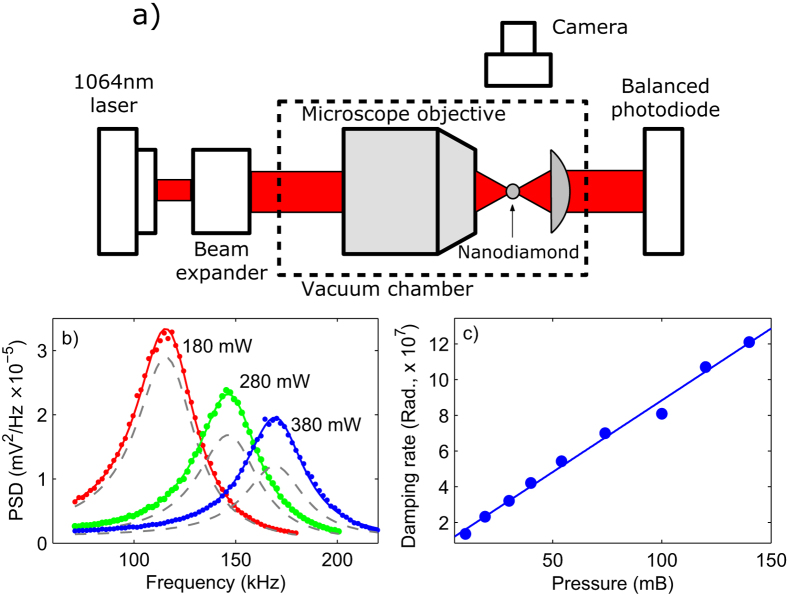
The trap was formed using a NA = 0.80 microscope objective and a 1064 nm laser. (**a**) Schematic of the experiment, and (**b**) power spectral densities (PSDs) at different trapping powers at 20 mB along with the respective theoretical (grey dashed lines) PSDs at room temperature (*T*_*CM*_ = 300 K). In generating theoretical PSDs, all parameters except the *T*_*CM*_s have been assumed identical to the measured PSDs. Numbers besides the PSDs denote the respective trapping power at the laser focus. (**c**) Blue circles are the measured damping rate (*γ*_*CM*_) as a function of pressure (*P*) with 180 mW of trapping power and the blue line is the linear fit.

Figure 1a shows a schematic of our experimental setup where we use a 0.80 numerical aperture (NA) microscope objective to focus a 1064 nm laser beam into a diffraction limited spot. The force resulting from the electric field gradient forms the basis of our dipole trap^20^. The balanced photodiode visible in Fig. 1a provides a voltage signal generated from the interference between the directly transmitted trapping laser light and the oscillator’s position dependent scattered electromagnetic radiation^20^. Performing a Fourier transform on this voltage signal provides the measured spectral information as well as the damping rate of a levitated nanoparticle. We use this spectral information and damping rate to retrieve *T*_*i*_ and the size of a nanodiamond.

In the regime where the oscillation amplitude of a trapped particle is small, the trapping potential of an optical tweezer can be approximated as harmonic^20^. Under this condition, the motion of a levitated object can be expressed as




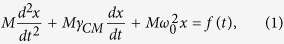




where *x* is the displacement of a trapped particle from the centre of the trap along the *x*-axis. *M* and *γ*_*CM*_, respectively, are the mass and the damping rate of a trapped particle while 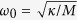 is the trap frequency and *κ* is the spring constant of the trap^20^. *f*(*t*) is a Gaussian random force exerted by the gas molecules on a trapped particle with 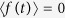 and 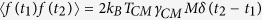, where *k*_*B*_ is the Boltzmann constant, *T*_*CM*_ is the CM temperature of a trapped particle, and *δ*(*t*_2_ − *t*_1_) is the Dirac delta function^20^. Similar analyses for the remaining two axes are also valid. After performing a Fourier transform and rearrangement, the power spectral density (PSD) of (1) can be written as




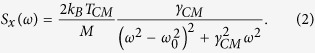




We fit (2) with the experimental data.

Figure 1b shows the PSDs corresponding to the measured voltage signals from a levitated nanodiamond for different trapping powers along with the respective fits (solid lines) of equation (2) at 20 mB. For the purpose of comparison, in Fig. 1b we have also included the relevant theoretical PSDs (dashed grey lines). In plotting the theoretical PSDs we have assumed that all parameters are identical to the measured PSDs except *T*_*CM*_ which has been taken equal to 300 K. Figure 1c demonstrates the measured damping rate as a function of pressure at a constant trapping power of 180 mW. Later, we use this damping rate to find the size of a nanoparticle.

## Levitated Nanodiamonds in Vacuum

**Figure 2:**
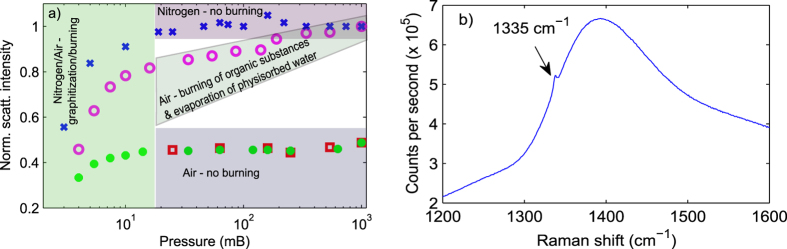
(**a**) Normalized scattering intensity as a function of pressure. Pink circles are for a nanodiamond as we take it to low pressures from atmospheric conditions for the first time and red squares are for the same nanodiamond but when we take it back to atmospheric pressure after keeping it at ≤10 mB for about an hour. Similarly, green dots are for the same nanodiamond used in the previous two steps but when we take it to low pressures for the 2^*nd*^ time from atmospheric pressure. Trapping power was 180 mW. Above 20 mB physisorbed water/organic substances evaporate/burn while below this pressure diamond or amorphous carbon burns. In the second round of evacuation a nanodiamond maintains its size down to ≈10 mB due to the absence of water and the organic substances on the surface. Blues crosses are the scattering intensities of a nanodiamond in a nitrogen environment. Trapping power was ≈300 mW. Down to 10 mB its size remains unchanged while below this pressure, due to elevated temperature, it graphitizes. (**b**) Raman spectrum of nanodiamonds under 785 nm laser excitation.

**Figure 3:**
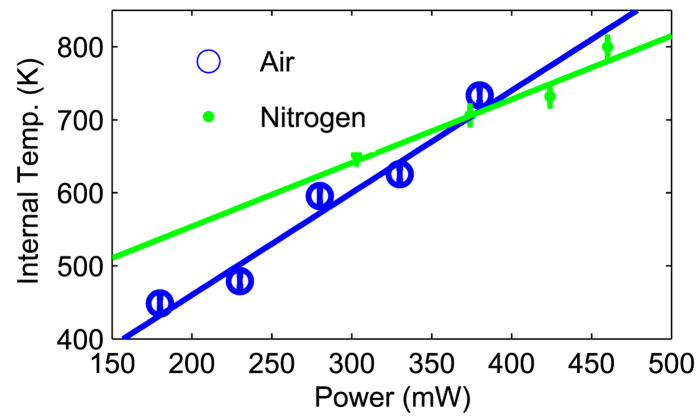
Internal temperature (*T*_*i*_) - blue circles in air and green dots in nitrogen at 20 mB as a function of trapping power. Solid blue and green lines represent linear fits.

To study the behaviour of diamond below atmospheric pressure, after levitating a nanodiamond with the minimum possible trapping power (180 mW), we gradually take it to different levels of vacuum whilst continuously monitoring its scattering intensity (size) using a camera. Figure 2a shows a typical plot of scattering intensity versus pressure (pink circles) from a levitated nanodiamond (for more data points see supplementary information Fig. S1). It can be observed that as we evacuate the trapping chamber, the scattering intensity diminishes: a levitated nanodiamond shrinks in size as the pressure is reduced. We attribute this reduction in size to the removal of physisorbed water and organic substances such as the carboxyl groups (nanodiamonds as obtained from the supplier are in water and are coated with carboxyl groups for stabilization) present on the surface of nanodiamonds down to 20 mB where the temperature reaches ≈450 K (see Fig. 3). Physisorbed water and organic impurities normally disappear^21^ at or below 473 K. This is further confirmed when we keep a levitated nanodiamond in a vacuum of less than 10 mB for an extended period of time (about an hour) and take it to back to atmospheric pressure (red squares in Fig. 2a) and bring it down to the low pressures again. In the second round of evacuation, the scattering intensity remains constant down to 10 mB. This unaltered scattering intensity in the second round of evacuation indicates the absence of substances which evaporate/burn at relatively lower temperatures.

The reduction in size below 10 mB is attributed to the burning of amorphous carbon or diamond. Amorphous carbon is generally found as an outer layer on the surface of nanodiamonds^21^,^22^,^23^. The burning temperature of amorphous carbon^21^ at atmospheric pressure varies between 573–723 K while the oxidation temperature of nanodiamonds^21^,^22^,^24^ ranges from 723–769 K. Also, the exact oxidation temperature of nanodiamonds depend on the surface quality, the crystallographic faces, and the densities of impurities in nanodiamonds^21^,^22^,^24^. To confirm the presence of amorphous carbon as well as diamond in the nanoparticles that we have used in our experiments, we performed Raman spectroscopy using a 785 nm laser. At this wavelength amorphous carbon is more sensitive than diamond^25^. Figure 2b presents the relevant data. This figure clearly shows the presence of amorphous carbon and diamond peaked at ≈1400 cm^−1^ and at ≈1335 cm^−1^, respectively^23^,^25^,^26^,^27^. Given that amorphous carbon is a strongly absorbing material^28^,^29^,^30^,^31^, trapping light (1064 nm) absorption and hence raised *T*_*i*_ and consequent burning in an air environment is highly probable. This burning of nanodiamond in air can potentially be a major hurdle in applications where vacuum is inevitable.

Based on the idea that an oxygen-less environment may be a cure to this problem, we have studied the behaviour of levitated nanodiamonds in a nitrogen environment. This is shown in Fig. 2a as blue crosses for a constant trapping power of 300 mW. It can be observed that at pressures >10 mB the scattering intensity hence the size of a nanodiamond remains unchanged; even though temperature is quite high (see Fig. 3). This is due to the fact that for burning to occur, a nanodiamond requires oxygen which is absent in a nitrogen rich environment. However, if the pressure is reduced below 10 mB, the scattering intensity of the nanodiamond gradually diminishes. Given that there is almost no oxygen in the chamber and the reduced pressure means less cooling due to gas molecules and hence higher internal temperature, we believe this is the onset of graphitization of the nanodiamond. At atmospheric pressure graphitization of nanodiamonds starts in the temperature range 943–1073 K and depends on the surface quality of nanodiamonds^24^,^26^. Since we are operating at sub-atmospheric pressures, graphitization at a lower temperature is most likely to happen. Lastly, it is noteworthy that irrespective of an air or a nitrogen environment, below 5 mB levitated nanodiamonds rapidly shrink in size and by ≈2 mB completely disappear from the trap.

## Internal Temperature of a Levitated Nanodiamond

**Figure 4:**
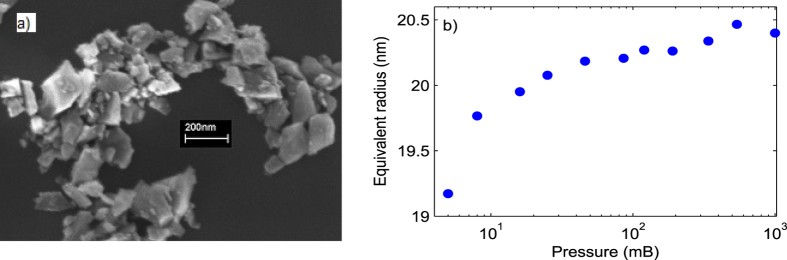
(**a**) Scanning electron microscope image of nanodiamonds as received from Adamas Nanotechnologis Inc., USA, and (**b**) the equivalent radius using equations (3) and (4) of the trapped nanodiamond for which the internal temperatures were found in Fig. 3 in air as a function of pressure.

Even though the nanodiamonds that we use in our experiments contain NV^−^ centres, most of them do not fluoresce upon levitation - consistent with the results of a previous study^32^ by Neukirch *et al.* It has been shown that the resonant frequency of optically-detected magnetic resonance from the fluorescing levitated nanodiamonds can reveal the internal temperature^32^, but in this article we instead use a Brownian motion based temperature determination technique developed by Millen *et al.* in ref. 33. According to this technique, the interaction between two thermal baths - one consisting of the impinging gas molecules while the other is composed of the emerging gas molecules, is mediated by a levitated object whose internal temperature is higher than that of the impinging gas molecules. The temperature of the impinging gas molecules is *T*_*imp*_ while that of the emerging gas molecules is *T*_*em*_. *T*_*CM*_ can be expressed as 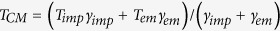, where *γ*_*imp*_ and *γ*_*em*_ are the damping rates due to the impinging and emerging gas molecules, respectively^33^. Using this methodology and assuming a full accommodation (*T*_*i*_ = *T*_*em*_), in Fig. 3 we present *T*_*i*_ obtained from the same nanodiamond used in Fig. 2 as a function of trapping power in air (blue circles) at 20 mB. In measuring *T*_*i*_ we have assumed that a levitated nanodiamond is at room temperature at ≈150 mB (see supplementary info Fig. S2). This assumption is also supported by the optically detected magnetic resonance based temperature measurements performed on nanodiamonds by Hoang *et al.* using a similar setup to ours^34^. Also, since fitting uncertainties increase with the increasing pressure, *T*_*i*_ has been plotted as a function of trapping power at a constant pressure and it was measured during the 2^*nd*^ round of evacuation at which a levitated nanodiamond maintains its size. Constancy in size/mass is a requirement of the PSD analysis. From Fig. 3 one can see that the internal temperature reaches ≈750 K at 380 mW of trapping power in air. This is well within the reported burning temperature of amorphous carbon or diamond^21^,^22^,^24^. In Fig. 3 we have also included *T*_*i*_s obtained from a levitated nanodiamond submerged in a nitrogen environment. In this case *T*_*i*_ reaches approximately 800 K at the maximum trapping power. At pressures below 20 mB, temperatures are expected to be higher given that the cooling due to gas molecules becomes less effective while the absorption remains constant. It is noteworthy that the fluorescence from NV^−^ centres in diamond decreases significantly at temperatures beyond 550 K and by 700 K it reduces to 20% of the room temperature value^13^. Also, at *T*_*i*_ = 700 K, NV^−^ centre’s fluorescence lifetime and the contrast between electron spin resonances reduce below 20% of the room temperature value^13^. At a temperature above 625 K, the spin coherence time of the NV^−^ centre decreases as well^13^. Furthermore, the highest temperature that we have measured here, using trapping powers higher than those have been used by Neukirch *et al.*^32^, rules out the possibility of melting diamond as suggested in ref. 32. Diamond usually melts at temperatures ≥4000 K and requires pressure above atmospheric pressure^35^. A slight difference between the temperatures at a constant power such as at 300 mW in Fig. 3 between different environments can be attributed to the variation in surface qualities and the densities of impurities in different nanodiamonds^24^,^36^. Additionally, it has been demonstrated that bigger particles heat up rapidly compared to smaller particles under the same experimental conditions^33^. As a result, variation in the internal temperatures is expected unless all the attributes of different particles are identical. However, due to the inherent nature of levitated experiments, it is difficult to levitate particles with the same attributes in different runs of an experiment. This is further worsened by the polydispersity of nanoparticles. For example, the average size of the nanodiamonds that we have used in our experiment is quoted to be 100 nm by the manufacturer. A representative scanning electron microscope (SEM) image of this nanodiamond is shown in Fig. 4a. Nanodiamonds from a few tens of nanometers to a few hundred nanometers are visible. Consequently, trapping different sizes of nanodiamonds in different runs of an experiment is possible. Nevertheless, to be consistent throughout the experiment, we levitate nanodiamonds of similar size by monitoring their scattering intensities. Also, next we present a way of determining the size of an individual levitated object from the measured damping rate (*γ*_*CM*_) that it encounters while oscillating inside the trap. For the purpose of following calculations, we assume that a levitated nanodiamond is of spherical shape.

## Determination of the Size of a Levitated Nanodiamond

The effective damping rate as shown in Fig. 1c can be expressed as *γ*_*CM*_ = *γ*_*imp*_ + *γ*_*em*_, where *γ*_*imp*_ and *γ*_*em*_ are the damping rates due to the impinging and emerging gas molecules, respectively^33^. *γ*_*imp*_ can be written as 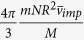 while *γ*_*em*_ is related to *γ*_*imp*_ by 
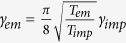
, where *R*, *N*, *m*, and 
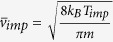
 are the radius of a trapped particle, the number density of gas molecules at pressure *P*, molecular mass, and the mean thermal velocity of impinging gas molecules, respectively^33^. *N* can further be expressed as *N* = *N*_0_*P*/*P*_0_, where *N*_0_ is the number of gas molecules per cubic meter at atmospheric conditions and *P*_0_ is the atmospheric pressure. On substitutions of various terms, one can express *R* as




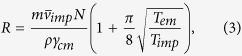




where *M* has been expressed as 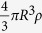 and *ρ* is the mass density of diamond.

Given that the levitated nanodiamonds burn, equation (3) gives the ultimate size of a nanodiamond for which we previously found temperatures. That is, it is the size of the nanodiamond after the first round of evacuation. The actual size of a nanodiamond before burning can be found using scattering theory. The scattering intensity of a Rayleigh particle 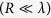 is given by 
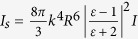
, where 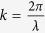 and *I* is the intensity of the trapping light^37^. Provided that we know the scattering intensity (see Fig. 2b) at different pressures, we can find the actual size of a nanodiamond using equation (4):




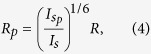




where *R*_*p*_ and 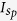 are the radius and the scattering intensity of the particle at pressure *P*, respectively.

As examples, using the model developed here, we estimate the sizes of the nanodiamonds for which we have presented internal temperatures in Fig. 3. Using equations (3) and (4), and parameters *N*_0_ = 2.43 × 10^25^, *T*_*imp*_ = 300 K, *T*_*em*_ = 450 K, *ρ* = 3500 kg/*m*^3^, *m* = 4.81 × 10^−26^ kg, *P* = 20 mB and *γ*_*cm*_ = 2.18 × 10^5^ radian with the minimum trapping power of 180 mW, Fig. 4b shows the radius of the trapped nanodiamond at various pressures in air. It can be observed that when the nanodiamond was initially trapped at atmospheric pressure, its diameter was ≈41 nm. Similarly, for the nitrogen case using the same parameters except *γ*_*cm*_ = 2.22 × 10^5^ radian and *T*_*i*_ = 650 K, we get the ultimate diameter of the nanodiamond is ≈38 nm. Given the uncertainty in the shape of nanodiamonds as visible in Fig. 4a, the nanodiamonds that we have used to find *T*_*i*_s in air and nitrogen ambients are of similar size. This is also in good agreement with the technique (initial scattering intensities) that we have utilized to trap similar size nanodiamonds in different runs of an experiment. Furthermore, even though the actual dimensions of a nanodiamond will be different from *R* due to its asymmetric shape, the estimated size provided by our model is well within the distribution visible in the SEM image (Fig. 4a). Lastly, we believe that the method developed here for the determination of size of an individual particle can be used in any levitated experiment.

## Conclusions

We have demonstrated that nanodiamonds burn in air while they graphitize in a nitrogen ambient by absorbing trapping laser (1064 nm) light as the cooling due to gas molecules becomes less effective with decreasing pressure. We believe that amorphous carbon, a strongly absorbing material, present on the surface of nanodiamonds is a key reason for this. We also think that purer nanodiamonds instead of the currently available HPHT synthesized nanodiamonds can be a cure to this problem. Our Brownian motion based analysis has shown that the internal temperature of a levitated nanodiamond can reach up to 800 K. This rules out the possibility of melting diamond which requires^35^ a temperature ≥4000 K. Lastly, exploiting the damping rate that a particle encounters while in motion, we have developed a new way of determining its size. We consider that this new technique will be useful in present and future levitated experiments where the traditional electron microscopy and dynamic light scattering based size determinations are not suitable.

## Methods

Nanodiamonds containing ≈500 NV^−^ centres (ND-NV-100 nm) were bought from Adamas Nanotechnology Inc, USA. The average size of the nanodiamonds quoted by the manufacturer is 100 nm. To prevent agglomeration we sonicate as received nanodiamonds for ≈10 minutes in an ultrasonic bath and then put them into a nebulizer and inject them into the trapping chamber. The trapping chamber is continuously monitored by a CMOS camera (Thorlabs Inc). Once a nanodiamond is trapped, the trapping chamber is evacuated to study the behaviour of nanodiamonds in vacuum. Power spectral density data were collected using a balanced photodiode (Thorlabs Inc) and a Picoscope oscilloscope (Pico Technology, UK). In the case of nanodiamonds immersed in nitrogen, the trapping chamber was purged with nitrogen fifteen times.

## Additional Information

**How to cite this article**: Rahman, A. T. M. A. *et al.* Burning and graphitization of optically levitated nanodiamonds in vacuum. *Sci. Rep.* **6**, 21633; doi: 10.1038/srep21633 (2016).

## Supplementary Material

Supplementary Information
