# Supplementary material for: Burning and graphitization of optically levitated nanodiamonds in vacuum
Source: Sci Rep. 2016 Feb 22;6:21633. doi: 10.1038/srep21633 (PMC4761924; doi:10.1038/srep21633)
Supplement: Supplementary Information [file srep21633-s1.pdf]

Burning and graphitization of optically levitated nanodiamonds in vacuum

A T M A. Rahman,[1,2] A. C. Frangeskou[2] , M. S. Kim,[3] S. Bose,[1] G. W. Morley,[2] and P. F. Barker[1]

[1]Department of Physics and Astronomy, University College London, Gower Street, WC1E 6BT, UK

[2]Department of Physics, University of Warwick, Gibbet Hill Road, CV4 7AL, UK

[3]QOLS, Blackett Laboratory, Imperial College London, SW7 2BW, UK

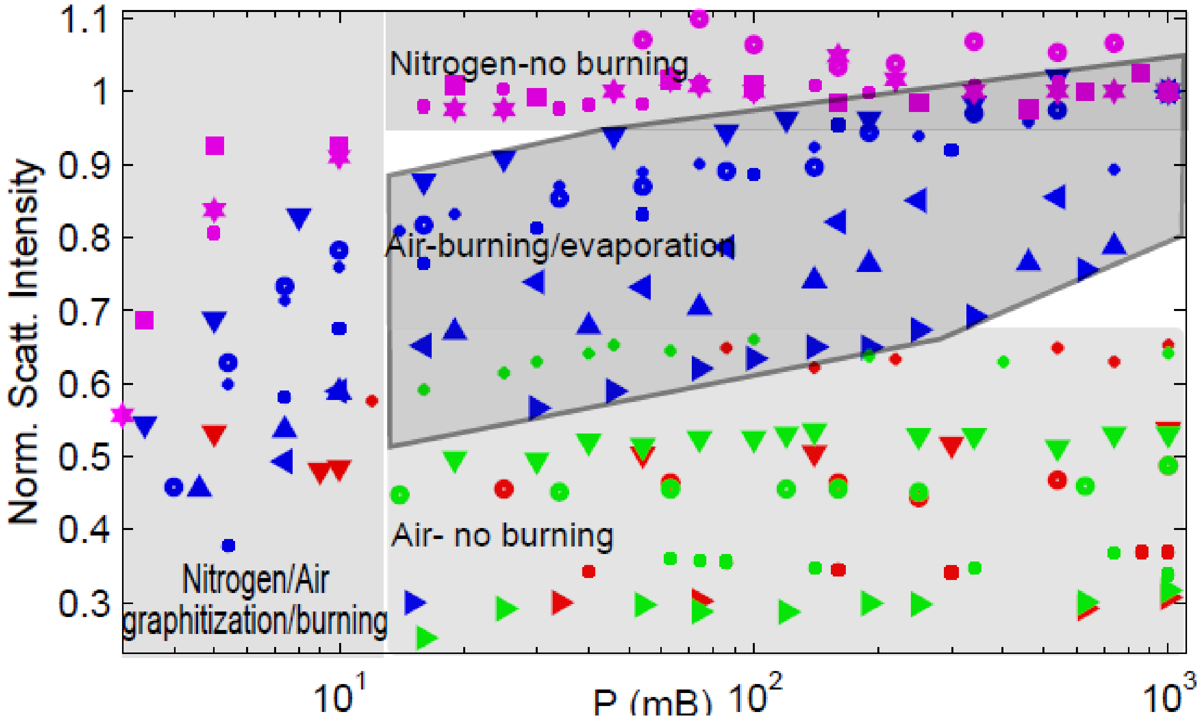

Figure – S1: Scattering intensity vs. pressure. Pink points are from levitated nanodiamonds under a nitrogen ambient while the blue points are from nanodiamonds in an air ambient. Data points corresponding to the red colours represent levitated nanodiamonds when we take them to atmospheric pressure after keeping them in a vacuum of ≤ 10 mB for about an hour. Similarly, data points in green show normalized scattering intensities when we take nanodiamonds to different levels of vacuum for the second time as described in the main text. Same maker such as a circle in blue, red and green represents scattering intensities from the same nanodiamond while we take it to vacuum for the first time after trapping, when we take it back to atmospheric pressure and when we bring it down to the low pressures for the second time, respectively.

Figure S1 shows additional data points in addition to Fig. 2a in the main text. The same trend as visible in the Fig. 2a in the main text is also evident here.

Even though at 150 mB fitting parameter related to the CM temperature increases slightly as we increase the laser power as shown in Figure S2, we assume that a trapped diamond is at room temperature when the trapping power is at the minimum. It is needed to remember that these are not absolute temperatures. To find absolute temperatures one needs to know the trap calibration factor C and mass of the particle M or one needs to assume a value of this parameter at a higher pressure as room temperature.

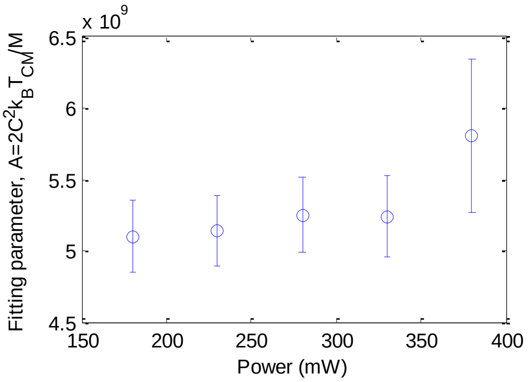

Figure 1: Fitting parameter containing CM temperature (T_{cm}), Boltzmann constant (k_B), calibration factor C and mass of the trapped particle M as a function of trapping power.
